# Predictive utility of a simple cranial magnetic resonance imaging score at term‐equivalent age for cerebral palsy

**DOI:** 10.1111/dmcn.70219

**Published:** 2026-03-16

**Authors:** Anne‐Kathrin Dathe, Britta Maria Huening, Pia Deborah Weber, Uta Teschler, Maire Brasseler, Ursula Felderhoff‐Mueser, Monia Vanessa Dewan

**Affiliations:** ^1^ Neonatology, Paediatric Intensive Care and Paediatric Neurology, Department of Paediatrics I University Hospital Essen, University of Duisburg‐Essen Essen Germany; ^2^ Centre for Translational Neuro‐ and Behavioural Sciences, C‐TNBS, Faculty of Medicine University of Duisburg‐Essen Essen Germany; ^3^ Department of Health and Nursing, Occupational Therapy Ernst‐Abbe‐University of Applied Sciences Jena Germany

## Abstract

**Aim:**

To evaluate the predictive value of the simple Total Abnormality Score (TAS) for assessing cranial magnetic resonance images to predict cerebral palsy (CP).

**Method:**

In this retrospective cohort study, 137 infants with a gestational age no more than 32 + 0 weeks and/or with a birthweight less than 1500 g, born between 2017 and 2021, were included. Findings from cranial magnetic resonance imaging at term‐equivalent age were assessed using the TAS. At approximately 24 months (corrected age), the diagnosis of CP and its severity using the Gross Motor Function Classification System (GMFCS) were determined interdisciplinarily. A binary logistic regression with CP as the outcome parameter and a Spearman's rank correlation with TAS and GMFCS were performed in SPSS.

**Results:**

The TAS was higher in infants with CP (median 11, interquartile range 10–15, *n* = 9) than in infants without CP (median 2, interquartile range 2–4, *n* = 128). The odds ratio for CP was 1.31 per one‐point increase in TAS. The optimal cut‐off value of the TAS for predicting CP was 9.5 (sensitivity 88.9%, specificity 91.4%). There was a correlation between the TAS and GMFCS (Spearman's rho = 0.34, *p* < 0.001).

**Interpretation:**

The TAS is a useful, quick tool for risk stratification of CP in this cohort. Future studies should consider replication in a larger sample and combination with additional tools.

## Graphical abstract

This study evaluated the Total Abnormality Score (TAS) from cMRI at term‐equivalent age as a predictor of cerebral palsy (CP) in 137 infants born preterm or with low birthweight. Infants who developed CP had significantly higher TAS values (median 11 vs 2), with a TAS cut‐off of 9.5 demonstrating high sensitivity (88.9%) and specificity (91.4%) for CP prediction. The TAS proved to be a simple, effective risk stratification tool, with scores correlating with motor function severity, though validation in larger cohorts is needed.

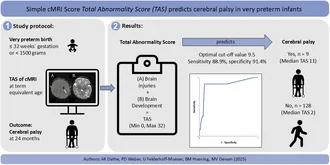

**This original article is commented by de Vries and Cowan on pages** 1641‐1642 **of this issue.**

**Abbreviations**
Bayley‐III: Bayley Scales of Infant and Toddler Development, Third Edition
BPD: bronchopulmonary dysplasia
CBH: cerebellar haemorrhage
IVH: intraventricular haemorrhage
PVL: periventricular leukomalacia
TAS: Total Abnormality Score
TEA: term‐equivalent age
WMI: white matter injury

**What this paper adds**

- Total Abnormality Score (TAS) predicts cerebral palsy in infants born very preterm at 24 months corrected age.
- The optimal cut‐off value of TAS for predicting cerebral palsy is 9.5.
- The correlation between TAS and the Gross Motor Function Classification System is of medium‐sized effect.
- TAS ≥9.5 predicts low cognitive and motor outcomes in the Bayley Scales of Infant and Toddler Development, Third Edition.

Preterm birth is the major risk factor for the development of cerebral palsy (CP).^1^, ^2^, ^3^ The risk of developing CP increases with decreasing gestational age. Infants born before 29 weeks' gestation are at the highest risk, with a prevalence of around 5% to 10%.^1^ Approximately half of all infants with CP are born preterm.^2^, ^3^ The revised definition of CP describes it as an early‐onset lifelong neurodevelopmental condition characterized by activity limitations due to impaired movement and posture development.^4^ It manifests as spasticity, dystonia, choreoathetosis, and/or ataxia, resulting from maldevelopment through dysplasia or injury to the fetal or infant brain (non‐degenerative, although manifestations may change with age). The CP phenotype is complex and heterogeneous. Beyond motor dysfunction, people with CP frequently encounter primary and secondary impairments across various developmental and functional areas (musculoskeletal system, sensation, feeding, communication, cognition, mental health, sleep, pain, and epilepsy) that can significantly affect participation in daily life. CP is further complicated by respiratory infections, aspiration‐related lung dysfunction, acquired scoliosis, and metabolic disorders (e.g. diabetes type 2, cardiovascular disease).^4^

A diagnosis of CP typically occurs between 12 to 24 months in high‐income countries.^5^, ^6^ However, an earlier diagnosis to introduce specific interventions during a window of high neuroplasticity to minimize restrictions on muscle tone, bone growth, and development is desirable.^7^ Following the international guidelines for early CP detection^7^ in infants with a corrected age of no more than 5 months, the most predictive tools are the General Movement Assessment (98% sensitivity), the Hammersmith Infant Neurological Examination (90% sensitivity),^7^, ^8^ and cranial magnetic resonance imaging (cMRI) at term‐equivalent age (TEA) (86%–89% sensitivity).^7^ In contrast to the evaluation of the General Movement Assessment and the Hammersmith Infant Neurological Examination, the evaluation of cMRI is less standardized. Moderate or severe white matter abnormalities, especially cystic white matter injury (WMI), are predictive of CP.^1^, ^9^ However, intraventricular haemorrhages (IVH), including low‐grade IVH as well as cerebellar haemorrhages (CBH), can also contribute to the manifestation of CP. Because the different types of brain injury can influence each other and might have a significant impact on brain development, a comprehensive evaluation of cMRI at TEA is necessary.^10^

For a structured evaluation of cMRI at TEA of infants born very preterm, most common scoring systems are useful.^1^, ^11^ However, these scores are time‐consuming for clinical routine (e.g. by including volumetry). The Total Abnormality Score (TAS) is an easily accessible and simple tool for clinicians for a structured evaluation of cMRI at TEA.^12^, ^13^ We recently showed that the TAS predicts the absence of fidgety movements at the corrected age of 3 months.^14^

The primary objective of this retrospective cohort study was to evaluate the predictive value of the TAS at TEA for the development of CP at 24 months corrected age and to determine an optimal cut‐off value for risk stratification. Secondary objectives included examining the correlation between the TAS and severity of CP as measured by the Gross Motor Function Classification System (GMFCS). Additionally, a subgroup analysis investigated the association between high or low TAS and cognitive and motor development.

## METHOD

### Participants

Infants born and/or treated in a level III neonatal intensive care unit between January 2017 and June 2021 with a gestational age of no more than 32 + 0 weeks and/or a birthweight less than 1500 g were included (Figure S1). Infants with congenital malformations or chromosomal anomalies were excluded. Infants without cMRI at TEA or missing follow‐up were excluded. Clinical data were retrieved from hospital records. Highest parental education was obtained by the International Standard Classification of Education.^15^ This study was performed in accordance with the Declaration of Helsinki and approved by the Ethics Committee of University of Duisburg‐Essen (15‐6522‐BO; 12–4981‐BO). The retrospective study design allows the use of pseudonymous data, eliminating the need for patient consent.

### Cerebral MRI acquisition

As part of routine care for infants born very preterm, cMRI was performed on a 3‐tesla MRI scanner (Skyra, Siemens Healthcare, Erlangen, Germany). Infants were examined in an MRI‐compatible incubator (Lammers Medical Technology nomag IC, Luebeck, Germany) using a dedicated eight‐channel neonatal head coil, in the presence of a paediatrician and a nurse. To minimize stress and noise, infants were swaddled, fed, and provided with an individual noise protection with a malleable paste (Affinis) and mini muffs (Natus Medical Incorporated, Seattle, WA, USA). MRI‐compatible pulse oxymetry (Invivo Essential, Orlando, FL, USA) was used to monitor heart rate and oxygen saturation levels. If necessary, oral sedation with chloral hydrate (25–50 mg/kg bodyweight) was administered through a gastric tube to avoid motion artefacts. The previously published standard imaging protocol^13^ consisted of transversal T2‐weighted turbo spin echo, T1‐weighted three‐dimensional fast low‐angle shot (FLASH), and susceptibility weighted and diffusion weighted (gradients: *b*_0_, *b*_700_, *b*_1000_) imaging. The cMRI scan typically lasted around 20 minutes.

### Cerebral MRI analysis: TAS

Two neonatologists (BMH, MVD), blinded to the clinical course and the radiological reports, scored cMRI data using the TAS^12^, ^13^ (Table S1). TAS separately evaluates brain injury (IVH, WMI, and CBH scores) for each hemisphere as well as brain development (myelination, gyration, ventricular dilatation). The scoring follows a hierarchical, aetiologically‐based approach, with lesions being scored only once. To distinguish between IVH and periventricular leukomalacia (PVL) scores, the following explanation is provided for the severest cases: an IVH score of 4 is assigned when parenchymal cysts with evidence of previous haemorrhage (visible on susceptibility‐weighted imaging sequences) are identified, indicating posthaemorrhagic cystic evolution following periventricular haemorrhagic infarction. A PVL score of 4 (cystic PVL in the right column) is assigned when we identify parenchymal cysts without evidence of haemorrhage, indicating primary ischaemic/hypoxic white matter injury. Cumulative scores range from 0 for optimal conditions to 32 for the most unfavourable outcomes. To increase reliability in detecting the smallest haemorrhages, susceptibility weighted imaging is used.^16^ TAS results were compared with radiological reports. In cases of discrepancy, results were discussed with a paediatric radiologist.

### Classification of CP

At a corrected age of at least 18 months, infants born very preterm underwent comprehensive follow‐up evaluation, including a neurological examination, medical history, and neurodevelopmental assessment. The diagnosis required (1) non‐progressive brain lesion/injury, evidenced by cMRI at TEA, and (2) delayed or atypical motor development at follow‐up,^4^ classified in GMFCS^17^ level I or higher. GMFCS is a five‐point, evidence‐based tool assessing gross motor function in infants, focusing on independent sitting, walking, and mobility. In cases without neurodevelopmental assessment (not performed, invalid), the interdisciplinary opinion on the absence or presence of CP (by a physician, physiotherapist, occupational therapist) was deemed sufficient. The primary outcome was CP diagnosis; the secondary outcome was GMFCS level. CP motor types were defined as follows.^4^, ^18^ (1) Spasticity: elevated velocity‐dependent muscle tone, pathological reflexes. (2) Dyskinesia: involuntary muscle contractions, distinct from dystonia and choreoathetosis. (3) Ataxia: impaired motor coordination in voluntary movements with abnormal force, rhythm, and accuracy. (4) Hypotonia: centrally mediated hypotonia, non‐progressive, and not attributable to genetic, neuromuscular, or metabolic disorders; hypotonia is not usually considered a distinct subtype, except in the Australian Cerebral Palsy Register. (5) Mixed type: absence of predominant clinical manifestation of other types.

After calculating the cut‐off value for the TAS, all infants with a value higher than the cut‐off and without CP were systematically re‐evaluated to ensure that no signs of CP had been overlooked.

### Cognitive and motor outcome

The German Version of the Bayley Scales of Infant and Toddler Development, Third Edition (Bayley‐III)^19^ was used for subgroup analyses, not for inclusion criteria. Cognitive and motor development were quantified using composite scores (norm‐referenced: mean = 100, standard deviation 15). Fine and gross motor skills used standard scores (mean = 10, standard deviation 3).^19^

### Calculation of sample size

Owing to the absence of an effect size for predicting CP by TAS, calculation of sample size is not feasible on this basis. However, the General Movement Assessment at 3 months corrected age has been shown to be a reliable predictor of CP, with a high sensitivity of 98% and specificity 91%.^7^, ^20^ The TAS has also been found to predict pathological general movements with an effect size of *f* ^2^ = 0.26.^14^ On this basis, estimation of a priori sample size was done using optimal design software (https://www.danielsoper.com/statcalc), assuming an alpha level of *p* = 0.05. The results indicated that a sample size of *n* = 32 would be required to achieve 80% power for logistic regression in predicting CP using the TAS.

### Statistical analysis

Statistical analyses were conducted using SPSS 29.0 (IBM Corp., Armonk, New York, USA). Descriptive data for included and excluded infants were presented as mean with standard deviation and counts (percentages), assuming normal distribution and adequate sample size. *χ* ^2^ and Student's *t*‐tests were used for comparisons.

The primary outcome was CP diagnosis. Descriptive statistics for infants with and without CP and were presented as medians (IQR) for continuous variables and counts (percentages) for categorical variables. Non‐parametric tests (Mann–Whitney *U* test and Fisher's exact test) were used owing to the expected low number of cases of CP. A *p*‐value less than 0.05 was considered significant. Effect sizes were calculated using the Psychometrica website (http://www.psychometrica.de/effect_size.html). Therefore, the test statistics for the Mann–Whitney *U* test (*z*, *N*, *n*_2_) were converted into an effect size (Cohen's *d*). Logistic regression estimated the odds ratio of CP with TAS. Receiver operating characteristic curves evaluated model performance. The optimal cut‐off value for predicting CP was determined using the Youden index and closest top‐left method. Positive and negative predictive values were calculated.

The secondary outcome was GMFCS level. A possible association between TAS and GMFCS was examined using Spearman's rank correlation and scatter plots.

For subgroup analysis, infants were stratified into low/high TAS groups. Median and IQR values were reported for each Bayley‐III score. A Mann–Whitney *U* test compared group differences. Scores below one standard deviation from the normative mean were considered low. Fisher's exact test compared proportions of low scores between groups. Logistic regression estimated odds ratios of high TAS associated with low scores.

## RESULTS

### Participants

A total of 122 infants born very preterm were excluded owing to missing cMRI data and follow‐up at a corrected age of at least 18 months. Excluded infants had significantly higher birthweight, length, and head circumference than included infants (*n* = 137), but did not differ in terms of sex, gestational age, other perinatal parameters, parental education, or presence of IVH or PVL (Table S2).

The study comprised a total of 137 infants born very preterm who had cMRI at TEA (median 40 weeks, IQR 40–40) and follow‐up for identification of CP (median 24 months, IQR 23–25). The clinical characteristics of infants born very preterm with (*n* = 9) and without (*n* = 128) CP are shown in Table 1. The groups did not differ with respect to sex, gestational age, birthweight, head circumference, and length. All mothers received two courses of antenatal betamethasone during pregnancy, except for 16 who had missing data. In the CP group, more infants were outborn (33.3%) compared with the non‐CP group (4.7%). There were no group differences in Caesarean section rates, APGAR scores at 5 minutes and 10 minutes, umbilical cord pH, and amniotic fluid infection. Conditions such as moderate and severe bronchopulmonary dysplasia (BPD), treated BPD, pneumothorax, postnatal sepsis, treated patent ductus arteriosus, treated retinopathy of prematurity, necrotizing enterocolitis, focal intestinal perforation, and neonatal seizures did not differ between the groups. More infants with CP received surfactant and surgery in general. There were no significant differences in terms of treatment with catecholamines, hydrocortisone, or treatment of BPD, patent ductus arteriosus, and retinopathy of prematurity. The groups did not differ with respect to parental education.

**TABLE 1:** Clinical characteristics of infants born very preterm with (*n* = 9) and without (*n* = 128) CP.

	Infants without CP (*n* = 128)	Infants with CP (*n* = 9)	*p*
Female, *n* (%)	63 (49.2)	5 (55.6)	0.75
Median gestational age at birth (IQR), weeks	29.7 (27.3–31.0)	27.6 (24.7–30.6)	0.123
Median birthweight (IQR), grams	1135 (842–1465)	1100 (520–1288)	0.255
SGA, *n* (%)	7 (5.5)	2 (22.2)	0.109
Median length at birth (IQR), centimetres	37 (34–40)	36.0 (31–40)	0.340
Median head circumference at birth (IQR), centimetres	27.0 (24.0–28.4)	25.0 (20.8–27.0)	0.116
Antenatal betamethasone (two applications at intervals of 24 hours), yes, *n* (%)	116 (100) (*n* = 116)	7 (100) (*n* = 7)	1.0
Inborn, yes, *n* (%)	122 (95.3)	6 (66.7)	**0.014**
Caesarean section, yes, *n* (%)	114 (89.1)	7 (77.8)	0.283
Median 5‐minute APGAR score (IQR)	8 (7–9)	8 (7–8)	0.086
Median 10‐minute APGAR score (IQR)	9 (8–9)	8 (8–9)	0.227
Median umbilical cord pH (IQR)	7.32 (7.27–7.36) (*n* = 123)	7.29 (7.18–7.40)	0.548
BPD moderate/severe, yes, *n* (%)	14 (10.9)	2 (22.2)	0.310
Treated BPD, yes, *n* (%)	15 (11.7)	2 (22.2)	0.283
Hydrocortisone, yes, *n* (%)	17 (13.3)	2 (22.2)	0.612
Pneumothorax, yes, *n* (%)	8 (6.3) (*n* = 127)	0 (0)	1.0
Amniotic fluid infection, yes, *n* (%)	6 (4.7)	0 (0)	1.0
Postnatal sepsis, yes, *n* (%)	51 (39.8)	4 (44.4)	1.0
Treated patent ductus arteriosus, yes, *n* (%)	47 (36.7)	5 (55.6)	0.300
Treated retinopathy of prematurity, yes, *n* (%)	8 (6.3)	1 (11.1)	0.468
Focal intestinal perforation, yes, *n* (%)	2 (1.6)	0 (0)	1.0
Necrotizing enterocolitis, yes, *n* (%)	4 (3.1)	1 (11.1)	0.292
Neonatal seizures, yes, *n* (%)	9 (7.0)	2 (22.1)	0.155
Surgeries in general, yes, *n* (%)	23 (18.0)	5 (55.6)	**0.018**
Surfactant, yes, *n* (%)	76 (61.3) (*n* = 124)	7 (100) (*n* = 7)	**0.047**
Catecholamines, yes, *n* (%)	13 (10.2) (*n* = 127)	3 (33.3)	0.073
Median parental ISCED (IQR)	4 (3–6) (*n* = 116)	4 (3–6) (*n* = 8)	0.415

*Note*: Data are presented as median (IQR) if not indicated otherwise. Descriptive statistics are based on *n* = 128 for the non‐CP group and *n* = 9 for the CP group; otherwise, the number of infants is indicated in brackets. Values in bold indicate *p* < 0.05. APGAR score consists of appearance, pulse, grimace, activity, and respiration. Treated BPD, hydrocortisone; treated patent ductus arteriosus, pharmacological and/or surgical treatment; treated retinopathy of prematurity, pharmacological treatment.

Abbreviations: BPD, bronchopulmonary dysplasia; CP, cerebral palsy; IQR, interquartile range; ISCED, International Standard Classification of Education; SGA, small for gestational age, with birthweight < third centile.

### TAS at TEA

All infants received cMRI at TEA. Their age, weight, and head circumference did not differ between the groups (Table 2). Examples of cMRI scans scored with TAS are depicted in Figures 1 and 2. The TAS differed between the groups in its total score, injury score, and developmental score, with higher values in infants with CP.

**TABLE 2:** Descriptive characteristics of TAS, subscores, and parameters in infants with (*n* = 9) and without (*n* = 128) cerebral palsy.

	Cohort (*n* = 137)	Infants without CP (*n* = 128)	Infants with CP (*n* = 9)	*p*
Gestational age at cMRI, median (IQR)	40.1 (40.0–40.3)	40.1 (40.0–40.3)	40.0 (39.9–40.1)	0.223
Median head circumference at cMRI (IQR), centimetres	34.5 (33.1–35.5)	34.5 (33.5–35.5)	33.0 (32.0–34.5) (*n* = 7)	0.068 (*n* = 134)
IVH, yes, *n* (%)	33 (24.1)	29 (22.7)	4 (44.4)	0.219
IVH > grade II, yes, *n* (%)	12 (8.8)	8 (6.3)	4 (44.4)	**0.003**
CBH, yes, *n* (%)	20 (14.6)	18 (14.1)	2 (22.2)	0.619
CBH > grade II, yes, *n* (%)	5 (3.6)	4 (3.1)	1 (11.1)	0.292
White matter injury, yes, *n* (%)	26 (19.0)	20 (15.6)	6 (66.6)	**0.002**
Cystic PVL, yes, *n* (%)	13 (9.5)	8 (6.3)	5 (55.6)	**< 0.001**
Median TAS (IQR)	2 (2–6)	2 (2–4)	11 (10–15)	**< 0.001**
Median injury score (IQR)	0 (0–4)	0 (0–2)	8 (7–11)	**< 0.001**
Median IVH score (IQR)	0 (0–0)	0 (0–0)	0 (0–7)	0.051
Median CBH score (IQR)	0 (0–0)	0 (0–0)	0 (0–1)	0.385
Median white matter injury score (IQR)	0 (0–0)	0 (0–0)	8 (0–8)	**< 0.001**
Median developmental score (IQR)	2 (2–2)	2 (2–2)	3 (2–6)	**0.003**
Median myelination (IQR)	0 (0–0)	0 (0–0)	0 (0–1)	**0.026**
Median gyration (IQR)	1 (1–1)	1 (1–1)	1 (1–2)	**0.015**
Median ventricular dilatation (IQR)	1 (1–1)	1 (1–1)	1 (1–3)	**0.002**

*Note*: The TAS consists of injury score (IVH score + CBH score + white matter injury score) and developmental score (myelination, gyration, and ventricular dilatation). Values in bold indicate *p* < 0.05.

Abbreviations: CBH, cerebellar haemorrhage; cMRI, cranial magnetic resonance imaging; CP, cerebral palsy; IQR, interquartile range; IVH, intraventricular haemorrhage; PVL, periventricular leukomalacia; TAS, Total Abnormality Score.

**FIGURE 1:**
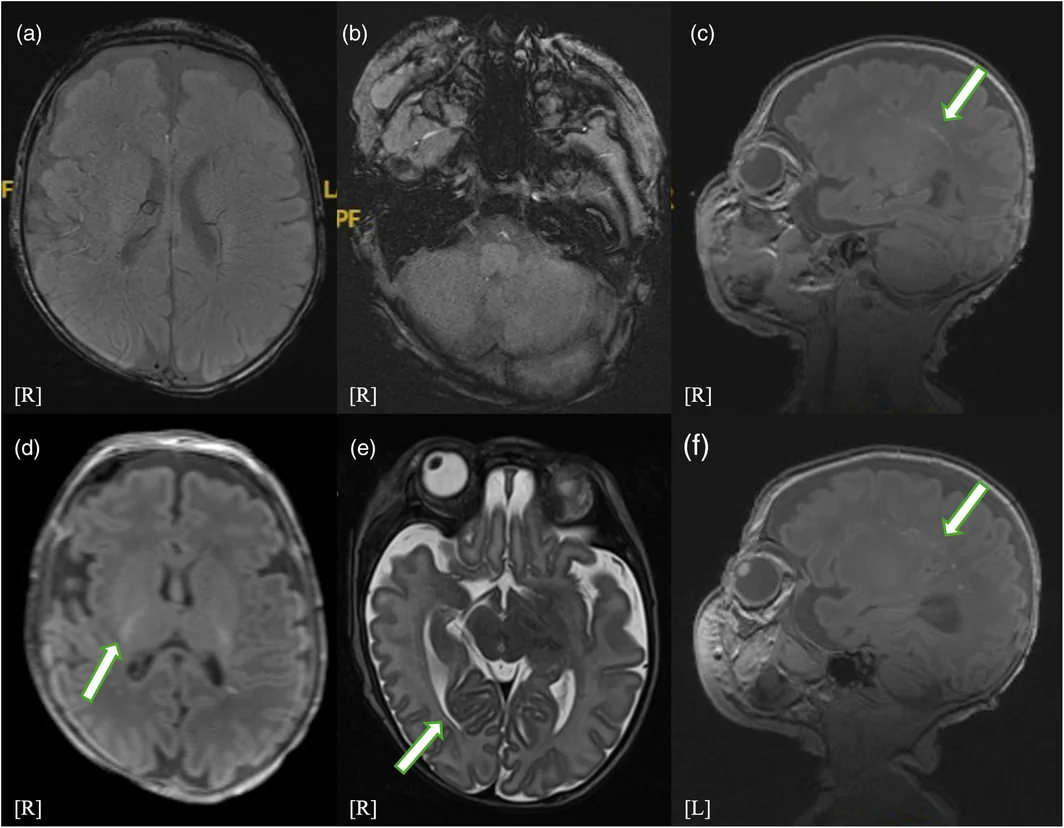
Brain magnetic resonance scan performed at term‐equivalent age in an infant born preterm at 31 + 1 weeks' gestation (infant 5 in Table S3) and corresponding TAS. TAS = 10 (injury score = 8, developmental score = 2). (a,b) Axial SWI with no signs of IVH or CBH (IVH and CBH score = 0). (c,f) Sagittal T1‐weighted imaging showing bilateral cluster of punctate lesions indicated by arrows (WMI score left = 4, right = 4). (d) Axial T1‐weighted imaging showing myelinated PLIC (arrow) (myelination = 0). (e) Axial T2‐weighted imaging showing sulci abutting the ventricles (arrow) (ventricular dilatation = 1). Gyration was scored 1, corresponding to 36–38 weeks' gestation (not shown). The infant achieved a cognitive composite score of 71 and a motor composite score of 55 in the Bayley Scales of Infant and Toddler Development, Third Edition. Abbreviations: CBH, cerebellar haemorrhage; IVH, intraventricular haemorrhage; [L], left; PLIC, posterior limb of internal capsule; [R], right; SWI, susceptibility weighted imaging; TAS, Total Abnormality Score; WMI, white matter injury.

**FIGURE 2:**
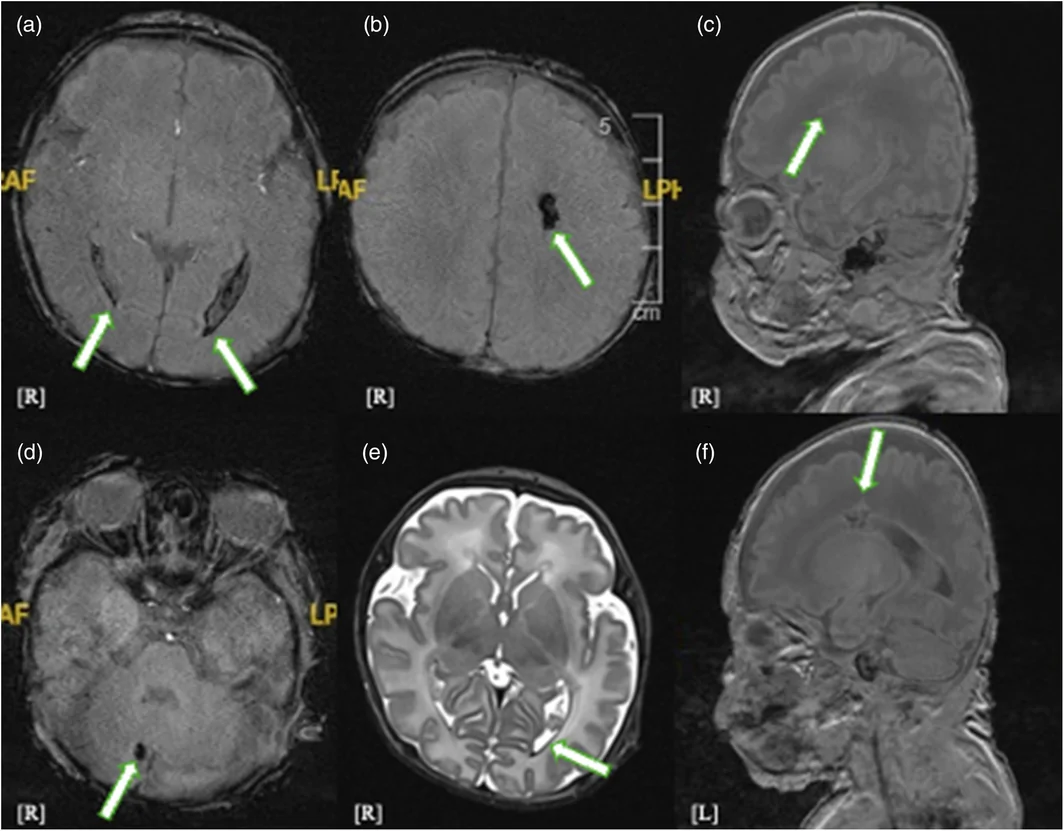
Brain magnetic resonance scan performed at term‐equivalent age in in an infant born preterm at 27 + 0 weeks' gestation (infant 6 in Table S4) and corresponding TAS. TAS = 18 (injury score = 16; developmental score = 2). (a,b) Axial SWI showing bilateral IVH indicated by arrows (IVH score left = 4, right = 2), (c,f) Sagittal T1‐weighted imaging showing bilateral cluster of punctate lesions (in addition to the posthaemorrhagic cystic lesion on the left; WMI score left = 4, right = 4). (d) Axial SWI showing cerebellar punctate haemorrhage (CBH score left = 0, right = 2). (e) Axial T2‐weighted imaging showing sulci abutting the ventricles (ventricular dilatation = 1). Myelination was scored 0; gyration was scored 1, corresponding to 36–38 weeks gestation (not shown). The infant achieved a cognitive composite score of 70 and a motor composite score of 79 in the Bayley Scales of Infant and Toddler Development, Third Edition. Abbreviations: CBH, cerebellar haemorrhage; IVH, intraventricular haemorrhage; [L], left; [R], right; SWI, susceptibility weighted imaging; TAS, Total Abnormality Score; WMI, white matter injury.

A significant difference was identified in white matter score between the groups. No differences were found in IVH or CBH scores. Infants with CP showed significantly higher scores in myelination, gyration, and ventricular dilatation, although medians were similar (Table 2).

Analysis of effect size revealed large effects between the CP and non‐CP groups for TAS (*d* = 0.714), injury score (*d* = 0.767), developmental score (*d* = 0.527), white matter score (*d* = 0.781), and ventricular dilatation (*d* = 0.542). Medium effects were observed for gyration (*d* = 0.425), IVH score (*d* = 0.339), and myelination (*d* = 0.339). CBH score showed a small effect (*d* = 0.149). On cMRI, there was a significant difference in WMI (non‐CP group 15.6%, CP group 66.6%).

### CP

Comprehensive details about CP and TAS can be found in Table S3. The sample included six infants diagnosed with spastic CP (five infants with bilateral impairments), one with mixed CP (distal spasticity, proximal hypotonia), one with ataxic CP, and one with hypotonic CP. The median GMFCS level was III (IQR II–IV) and a medium effect was observed in the correlation between TAS and GMFCS (Spearman's rho = 0.340, 95% confidence interval [CI] 0.178–0.484, *p* < 0.001) (Figure 3).

**FIGURE 3:**
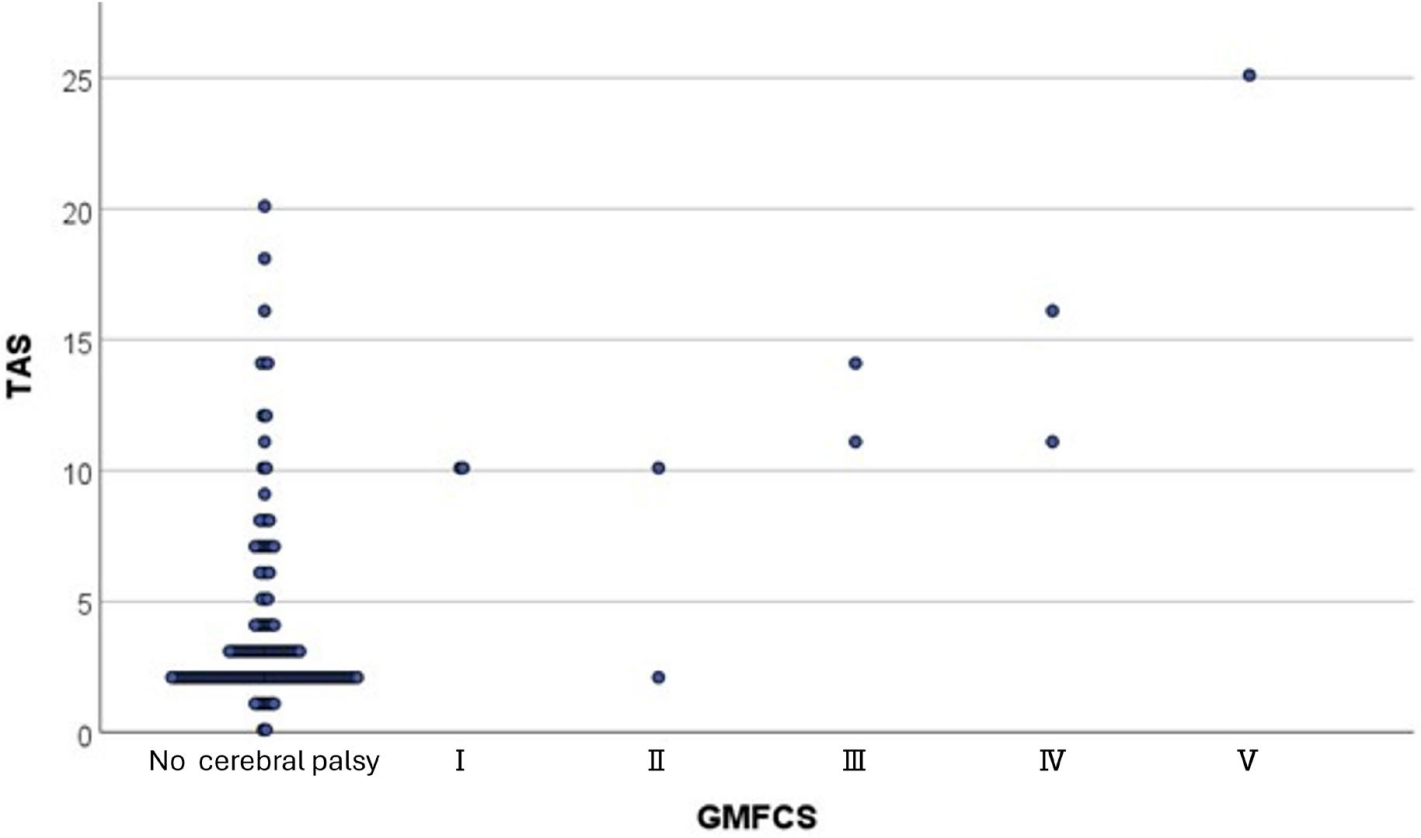
Scatterplot of Total Abnormality Score (TAS) and Gross Motor Function Classification Scale (GMFCS) (*n* = 137).

### Prediction of CP by TAS

A significant association between TAS and CP was confirmed, with an odds ratio for CP of 1.31 (95% CI 1.14–1.50) per one‐point increase in TAS (*χ* ^2^_1_ = 15.09, *p* < 0.001, Nagelkerke's *R* ^2^ = 0.34). The receiver operating characteristic curve for risk of CP by TAS (Figure 4) had an area under the curve of 0.87 (95% CI 0.74–1.00). The optimal cut‐off value of the TAS, according to the Youden index and the closest top‐left method, was 9.5 (sensitivity 88.9%, 95% CI 59.5–99.3; specificity 91.4%, 95% CI 85.7–95.4). Using a cut‐off value of 9.5 points on the TAS, the negative and positive predictive values were 99.2% (95% CI 96.3–100.0) and 42.1% (95% CI 22.0–64.2) respectively (cross‐tabulation in Table 3).

**FIGURE 4:**
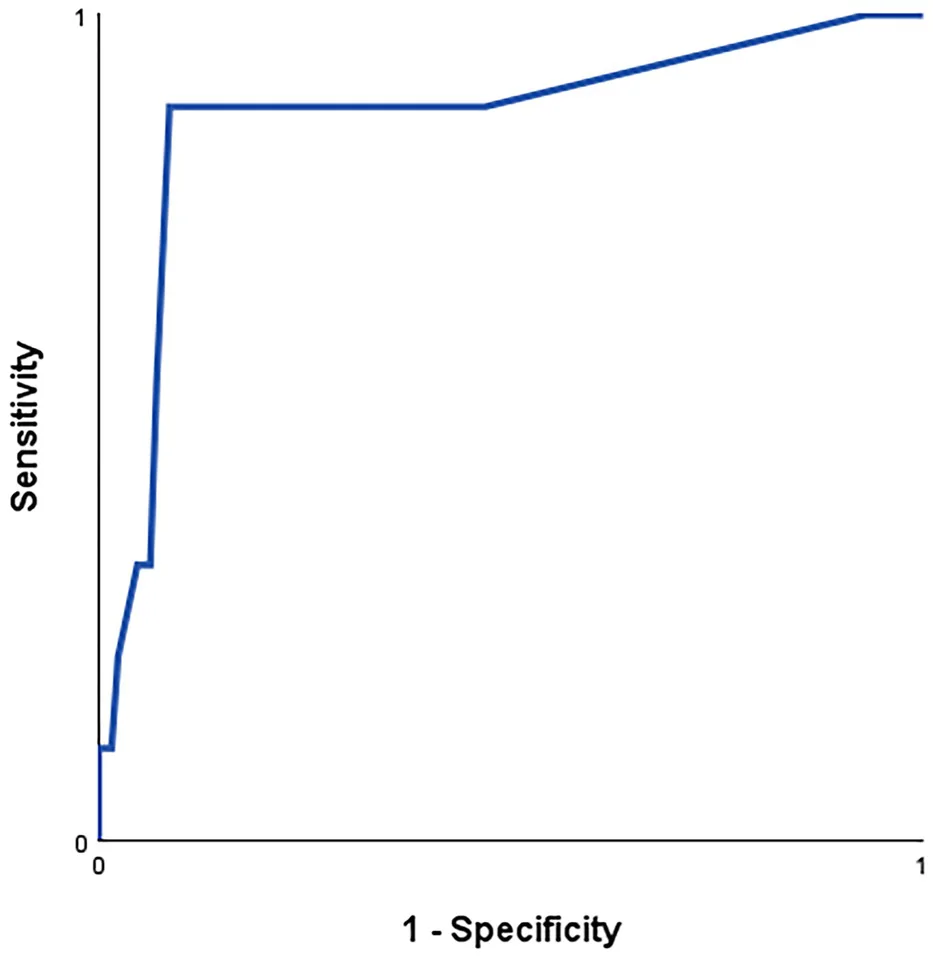
Receiver operating characteristics curve for Total Abnormality Score to predict cerebral palsy (*n* = 137).

**TABLE 3:** Cross‐tabulation of TAS cut‐off value of 9.5 in infants with (*n* = 9) and without (*n* = 128) cerebral palsy.

		No cerebral palsy, *n*	Cerebral palsy, *n*	Total, *n*
TAS ≥9.5	No	117	1	118
	Yes	11	8	19
Total		128	9	137

Abbreviation: TAS, Total Abnormality Score.

### Cognitive and motor development

A TAS of at least 9.5 was identified in 19 infants, eight of whom developed CP. Infants with a high TAS (≥9.5) had significantly lower cognitive and motor composite scores, and gross motor standard scores, but not fine motor scores. A greater proportion of infants with a high TAS had low scores (less than one standard deviation below the normative mean) in cognitive, motor composite, and gross motor scores than infants with a low TAS. Odds of low scores were significantly greater with a high TAS: cognitive composite odds ratio 5.71 (95% CI 1.95–16.79, *p* = 0.002), motor composite odds ratio 4.20 (95% CI = 1.40–12.60, *p* = 0.011), and gross motor odds ratio 5.62 (95% CI = 1.86–16.98, *p* = 0.002) (Tables 4, S3, and S4).

**TABLE 4:** Cognitive and motor outcomes in Bayley‐III in infants with low and high TAS.

	Infants with low TAS (<9.5) (*n* = 118)	Infants with high TAS (≥9.5) (*n* = 19)	*p*
Median corrected age (IQR), months	24 (23–24) (*n* = 108)	24 (23–24) (*n* = 17)	**0.596**
Median cognitive composite score (IQR)	100 (85–110) (*n* = 105)	80 (65–100) (*n* = 17)	**0.013**
Low cognitive composite score, yes, *n* (%)	21 (20.0)	10 (58.8)	**0.002**
Median motor composite score (IQR)	94 (82–104) (*n* = 102)	76 (61–91) (*n* = 16)	**0.004**
Low motor composite score, yes, *n* (%)	29 (28.4)	10 (62.5)	**0.010**
Median fine motor standard score (IQR)	10 (7–12) (*n* = 108)	8 (6–11) (*n* = 17)	0.168
Low fine motor standard score, yes, *n* (%)	17 (15.7)	4 (23.5)	0.485
Median gross motor standard score (IQR)	9 (7–10) (*n* = 102)	4 (1–8) (*n* = 16)	**< 0.001**
Low gross motor standard score, yes, *n* (%)	19 (18.6)	9 (56.3)	**0.003**

*Note*: Low scores are defined as scores lower than one standard deviation below the mean of the standard population. Data are presented as median (IQR) if not indicated otherwise. Values in bold indicate *p* < 0.05. Descriptive statistics are based on *n* = 118 for infants with low TAS and *n* = 19 for infants with high TAS; otherwise, the number of cases is indicated in brackets.

Abbreviations: Bayley‐III, Bayley Scales of Infant and Toddler Development, Third Edition; IQR, interquartile range; TAS, Total Abnormality Score.

## DISCUSSION

The aim of this study was to investigate the predictive value of the TAS for CP in infants born very preterm. The TAS at TEA was a good predictor of CP, with a sensitivity of 88.9% and specificity of 91.4%. Infants with CP exhibited significantly higher total, injury, and developmental scores. A one‐point increase in TAS was associated with 1.31‐fold increased odds of CP. Furthermore, a significant association was observed between TAS and GMFCS level with medium effect size. The optimal predictive cut‐off for CP was 9.5, with an excellent negative predictive value (99.2%) but poor positive predictive value (42.1%). Notably, one infant with a low TAS (<9.5) had CP. MRI at TEA is important for early diagnosis of CP.^7^ For infants born preterm, the positive predictive value for CP or low motor score on the Bayley‐III ranges between 60% and 67%.^21^ A structured global evaluation of brain alterations can improve the predictive value. The scoring system by Woodward et al. consists of a white and grey matter score with eight assessments on a three‐point scale. The score was applied on a cohort of 167 infants born very preterm born between 1998 and 2000 and included 17 (10%) infants who developed CP. The presence of moderate to severe white matter abnormalities was predictive of CP with a sensitivity of 65% and a specificity of 84%, while the sensitivity and specificity of any white matter abnormality was reported as 94% and 31% respectively.^1^ IVH and CBH were not included in the score.

The predictive value of the global abnormality score was re‐evaluated in a cohort of 239 infants born extremely preterm between 2006 and 2012 (gestational age < 28 weeks).^22^ The global brain abnormality score evaluates white and grey matter injury as well as deep grey matter and cerebellar abnormalities.^11^ While the predictive value for CP was not mentioned, the score was inversely related to cognitive and motor outcomes. However, explained variances were small, referring to the relative low rate of moderate/severe brain injury and only six (2.5%) infants diagnosed with CP.^22^ A simplified scoring system for brain injury (IVH, PVL, CBH), supplemented by simple metrics, by Kidokoro et al.^11^ reported high‐grade IVH and PVL as risk factors for CP development. In our study, significant differences between the CP and non‐CP groups were observed in WMI (66.6% vs 15.6% respectively) and in the white matter developmental score (medians 8 vs 0). These findings emphasize the importance of WMI in the pathogenesis of CP. Although the incidence of both cystic and non‐cystic WMI has declined, it remains the predominant entity of brain injury due to the vulnerability of immature oligodendrocytes.^23^ Although IVH or CBH are associated with the development of CP, the presence of these forms of brain injury per se did not differ significantly between the CP and non‐CP groups (IVH all grades 44.4% vs 22.7%; CBH all grades 22.2% vs 14.1%). Significantly more infants with IVH greater than grade 2 and periventricular haemorrhagic infarction had CP, as known risk factors due to the associated (or consequent) WMI.^10^

Although CBH is associated with CP, concomitant supratentorial injuries as confounding factors complicate the interpretation of study results.^10^ Studies focusing on isolated CBH could show that large size and localization are risk factors for severe motor delay.^24^ However, punctate lesions rather affect the cognitive and affective development.^25^ In our study, more infants had cerebellar punctate haemorrhagic lesions than a CBH greater than grade 2 (10.9% vs 3.6%).

Significant differences between the CP and non‐CP groups were observed in four out of six TAS parameters in our cohort. However, analysis of effect sizes revealed small (CBH score), medium (gyration, IVH score, myelination), and large (ventricular dilatation) effects. It is evident that a larger sample size is required to facilitate interpretation of individual effects of parameters for predicting CP. Owing to the low positive predictive value, the TAS is not suitable as a sole biomarker for CP. We recommend the use of additional assessments, such as the General Movement Assessment and the Hammersmith Infant Neurological Examination, to improve diagnostic accuracy.^7^ The only infant with CP and a TAS of 2 had a severe BPD in a difficult social environment. BPD is a known independent risk factor for the development of CP,^26^ as well as socioeconomic background.^27^ On the other hand, 11 infants with a TAS of at least 9.5 did not develop CP (e.g. the patient depicted in Figure 2). Subgroup analysis of infants with high and low TAS revealed significant differences in cognitive and motor development. TAS reliably predicted cognitive and motor development, predicting both CP and accompanying cognitive deficits. TAS associated with gross motor but not fine motor skills, possibly because fine motor impairments are less severe or become apparent later with increasing task complexity. The clinical utility of early MRI/TAS assessment thus extends beyond CP prediction to encompass identification of children requiring developmental support across multiple domains.

One 24‐month‐old infant with persistent central hypotonia was classified as having hypotonic CP, on the basis of clinical criteria and the absence of attributable progressive, genetic, or neuromuscular disorders. Although the Surveillance of Cerebral Palsy in Europe (2017) does not recognize hypotonia as a distinct subtype, its inclusion reflects the heterogeneity and may represent a variant or transitional form.

Since some cases with a TAS of at least 9.5 did not develop CP, microstructural MRI changes cannot be excluded and lesion topography for specific white matter tracts is limited. Advanced MRI techniques such as diffusion tensor imaging are needed for accurate evaluation of white matter tract^28^ but are not clinically routine owing to complex analyses. A multifactorial model including TAS and clinical and protective factors might improve the prediction accuracy for CP.

Study limitations include the small cohort, with a CP rate of 7.2%, although this is within the expected range.^1^ Additionally, the cut‐off value was determined from unadjusted logistic regression models. Given the limited sample size, particularly the low number of cases of CP, adjustment for multiple confounders would risk overfitting and unstable estimates. Future studies with larger samples should investigate whether adjustment for confounding variables such as gestational age, birthweight, or neonatal complications improves the predictive accuracy of the TAS. Multicentre, prospective replication with larger samples is essential to validate findings and investigate factors such as socioeconomic status and fetal influences.

The primary strength of this study lies in the comprehensive evaluation of the TAS using high‐quality cMRI with highly blood‐sensitive susceptibility‐weighted imaging sequences, enabling the detection of even the smallest haemorrhagic lesions. The cMRI scans were evaluated by a team of experienced paediatric radiologists/neuroradiologists and paediatricians/neonatologists, who were blinded to the clinical course.

## CONCLUSION

This study highlights the potential of the TAS as a simple tool for early detection of future severe neurodevelopmental impairments such as CP in infants born very preterm. Our findings demonstrate that the TAS is a reliable predictor of CP, with good sensitivity and excellent specificity. In addition, there was a significant association between TAS and the severity of CP measured with the GMFCS. Owing to the low negative predictive value, a combination of tools should be used to assess infants.

## FUNDING INFORMATION

Open Access Publication Fund of the University of Duisburg‐Essen.

## CONFLICT OF INTEREST STATEMENT

BMH acted as a consultant and lecturer for Chiesi GmbH. The other authors have stated that they had no interests that might be perceived as posing a conflict or bias.

## Supporting information


**Figure S1:** Participant flow and exclusion criteria.

**Table S1:** The Total Abnormality Score with subscores and parameters.

**Table S2:** Clinical characteristics of included and excluded infants born very preterm due to missing cMRI and follow‐up ≥18 months (corrected age).

**Table S3:** Detailed characteristics of infants with cerebral palsy.

**Table S4:** Detailed characteristics of infants with TAS ≥9.5 without cerebral palsy.

## Data Availability

The data that support the findings of the study are available on request from the corresponding author. The data are not publicly available owing to privacy or ethical restrictions.
