# Supplementary material for: Predictive utility of a simple cranial magnetic resonance imaging score at term‐equivalent age for cerebral palsy
Source: Dev Med Child Neurol. 2026 Mar 16;68(12):1729–39. doi: 10.1111/dmcn.70219 (PMC13743671; doi:10.1111/dmcn.70219)
Supplement: Supplementary file 1 [file DMCN-68-1729-s001.docx]

**Table S**3: Detailed Characteristics of infants with cerebral palsy (*n* = 9)

|  | Infant 1 | Infant 2 | Infant 3 | Infant 4 | Infant 5 | Infant 6 | Infant 7 | Infant 8 | Infant 9 |
| --- | --- | --- | --- | --- | --- | --- | --- | --- | --- |
| Female | no | no | no | yes | yes | no | yes | yes | yes |
| Injury Score | 13 | 8 | 8 | 6 | 8 | 0 | 8 | 19 | 8 |
| IVH grade left | 4 | 4 | 0 | 4 | 0 | 0 | 0 | 3 | 0 |
| IVH grade right | 4 | 4 | 0 | 2 | 0 | 0 | 0 | 2 | 0 |
| IVH Score | 8 | 8 | 0 | 6 | 0 | 0 | 0 | 5 | 0 |
| CBH grade left | 1 | 0 | 0 | 0 | 0 | 0 | 0 | 3 | 0 |
| CBH grade right | 1 | 0 | 0 | 0 | 0 | 0 | 0 | 3 | 0 |
| CBH Score | 2 | 0 | 0 | 0 | 0 | 0 | 0 | 6 | 0 |
| WMI left | 1 | 0 | 4 | 0 | 4 | 0 | 4 | 4 | 4 |
| WMI right | 2 | 0 | 4 | 0 | 4 | 0 | 4 | 4 | 4 |
| WMI Score | 3 | 0 | 8 | 0 | 8 | 0 | 8 | 8 | 8 |
| Developmental score | 3 | 6 | 2 | 5 | 2 | 2 | 3 | 6 | 2 |
| Myelination grade | 0 | 1 | 0 | 0 | 0 | 0 | 0 | 2 | 0 |
| Gyration | 1 | 2 | 1 | 1 | 1 | 1 | 2 | 2 | 1 |
| VD | 2 | 3 | 1 | 4 | 1 | 1 | 1 | 2 | 1 |
| TAS | 16 | 14 | 10 | 11 | 10 | 2 | 11 | 25 | 10 |
| CP, type | Spastic | Mixed | Ataxic | Spastic | Spastic | Spastic | Spastic | Hypotonic | Spastic, |
| CP, spasticity | Yes, bilateral | Yes, bilateral | No | Yes, unilateral | Yes, bilateral | Yes, bilateral | Yes, bilateral | No | Yes, bilateral |
| GMFCS at corrected 24 months | 4 | 3 | 2 | 3 | 1 | 2 | 4 | 3 | 1 |
| Parental ISCED | 2 | 6 | 3 | 3 | 3 | - | 4 | 6 | 6 |
| BSID |  |  |  |  |  |  |  |  |  |
| Cognitive composite score | - | 55 | 100 | 55 | 71 | 55 | 60 | 75 | 110 |
| Motor composite score | - | 49 | 61 | 61 | 55 | 49 | 49 | 73 | 100 |
| Fine motor standard score | - | 3 | 7 | 7 | 5 | 3 | 3 | 11 | 13 |
| Gross motor standard score | - | 1 | 1 | 1 | 1 | 1 | 1 | 1 | 7 |

Notes. IVH = intraventricular hemorrhage; CBH =cerebellar hemorrhage; WMI = grade

of white matter injury; VD = ventricular dilatation; CP = cerebral palsy, GMFCS = Gross motor function classification system; ISCED = international standard classification of education with low (1 and 2), middle (3 and 4), and high (> 4) level.
