# Supplementary material for: Predictive utility of a simple cranial magnetic resonance imaging score at term‐equivalent age for cerebral palsy
Source: Dev Med Child Neurol. 2026 Mar 16;68(12):1729–39. doi: 10.1111/dmcn.70219 (PMC13743671; doi:10.1111/dmcn.70219)
Supplement: Supplementary file 2 [file DMCN-68-1729-s003.docx]

**Table S1.** The Total Abnormality Score with subscores and parameters (13,14).

| **Injury Score^a^** | **Developmental Score^a^** |
| --- | --- |
| **IVH score (grades 0–4), (T1, T2, SWI)** 0 = no haemosiderin deposits 1 = haemosiderin deposits or post haemorrhagic cysts within caudo-thalamic notch 2 = haemosiderin deposits outside notch, along ventricle wall without VD 3 = obvious VD with evidence of previous ventricular haemorrhage  4 = parenchymal haemorrhagic lesions >3 mm or posthaemorrhagic cystic encephalopathy^b^ | **Myelination (grades 0–4), (T1)** 0 = ≥1/3 PLIC + centrum semiovale 1 = ≥1/3 PLIC + optic radiation, not centrum semiovale 2 = ≥1/3 PLIC, but not in centrum semiovale and optic radiation  3 = ≤1/3 PLIC, clearly visible 4 = no myelin in PLIC |
| **White matter injury score (grades 0–4), (T1, T2)** 0 = no signs for lesions in peri-ventricular WM on T1 and T2  1 = punctate lesions ≤3 mm and <3 lesions 2 = punctate lesions ≤ 3mm in corticospinal tract or punctate lesions ≤3 mm and ≥3 lesions in <3 regions (frontal, temporal, parietal, occipital) 3 = extensive lesions (>3 mm and/or >3 lesions in ≥3 regions)  4 = cystic lesions in periventricular WM or cluster (≥6 lesions) | **Gyration (grades 0–2), (T1)** 0 = 40 weeks, tertiary gyri/sulci inferior temporal and inferior occipital 1 = 36–38 weeks, additional secondary gyri/sulci transversi and inferior temporal 2 = 34–36 weeks, present marginal sulcus, paracentral gyrus, secondary gyri front. |
| **CBH score (grades 0–3), (T1, T2, SWI)** 0 = no haemorrhage 1 = haemorrhage ≤3 mm and ≤3 lesions 2 = punctate lesion(s) >3 mm and/or >3 lesions per hemisphere  3 = extensive lesions | **Ventricular dilatation (VD),**  **(grades 0–4), (T2)** 0 = slim ventricles both sides 1 = mild dilatation, but clear concave shape, with modelling to gyri  2 = moderate VD, borderline shape 3 = severe VD, rounded ventricular horns, convex shape 4 = porencephaly |

Notes. TAS = total abnormality score; PVL = periventricular leukomalacia; WM = white matter; IVH = intraventricular haemorrhage; VD = ventricular dilatation; SWI = susceptibility weighted imaging, CBH = cerebellar haemorrhage; PLIC = posterior limb of internal capsule; ^a^Injury and Developmental Scores are summed up to TAS. ^b^ = cystic evolution in an area of previous periventricular hemorrhagic infarction. Note that the Injury Score is obtained separately for the right and left hemisphere.
