# Supplementary material for: Predictive utility of a simple cranial magnetic resonance imaging score at term‐equivalent age for cerebral palsy
Source: Dev Med Child Neurol. 2026 Mar 16;68(12):1729–39. doi: 10.1111/dmcn.70219 (PMC13743671; doi:10.1111/dmcn.70219)
Supplement: Supplementary file 3 [file DMCN-68-1729-s004.docx]

**Table S**4: Detailed Characteristics of infants with TAS ≥ 9.5 without cerebral palsy (*n* = 11).

|  | Infant 1 | Infant 2 | Infant 3 | Infant 4 | Infant 5 | Infant 6 | Infant 7 | Infant 8 | Infant 9 | Infant 10 | Infant 11 |
| --- | --- | --- | --- | --- | --- | --- | --- | --- | --- | --- | --- |
| Female | Yes | Yes | Yes | No | Yes | No | No | Yes | No | Yes | Yes |
| Injury Score | 18 | 9 | 8 | 8 | 12 | 16 | 8 | 8 | 11 | 8 | 12 |
| IVH grade left | 4 | 2 | 0 | 4 | 2 | 4 | 4 | 2 | 4 | 0 | 4 |
| IVH grade right | 4 | 2 | 0 | 4 | 2 | 2 | 4 | 2 | 2 | 0 | 4 |
| IVH Score | 8 | 4 | 0 | 8 | 4 | 6 | 8 | 4 | 6 | 0 | 8 |
| CBH grade left | 2 | 2 | 0 | 0 | 0 | 0 | 0 | 0 | 0 | 0 | 0 |
| CBH grade right | 1 | 3 | 0 | 0 | 0 | 2 | 0 | 0 | 0 | 0 | 0 |
| CBH Score | 3 | 5 | 0 | 0 | 0 | 2 | 0 | 0 | 0 | 0 | 0 |
| WMI left | 4 | 0 | 4 | 0 | 4 | 4 | 0 | 4 | 4 | 4 | 4 |
| WMI right | 3 | 0 | 4 | 0 | 4 | 4 | 0 | 0 | 1 | 4 | 0 |
| WMI Score | 7 | 0 | 8 | 0 | 8 | 8 | 0 | 4 | 5 | 8 | 4 |
| Developmental score | 2 | 3 | 4 | 6 | 2 | 2 | 3 | 2 | 3 | 2 | 4 |
| Myelination grade | 0 | 0 | 1 | 0 | 0 | 0 | 0 | 0 | 0 | 0 | 0 |
| Gyration | 1 | 1 | 2 | 2 | 1 | 1 | 1 | 0 | 1 | 1 | 1 |
| VD | 1 | 2 | 1 | 4 | 1 | 1 | 2 | 1 | 2 | 1 | 3 |
| TAS | 20 | 12 | 12 | 14 | 14 | 18 | 11 | 10 | 14 | 10 | 16 |
| Parental ISCED | 3 | 3 | 4 | 7 | 4 | 4 | 3 | 3 | 7 | 6 | 8 |
| BSID |  |  |  |  |  |  |  |  |  |  |  |
| Cognitive composite score | - | 95 | 85 | 80 | 135 | 70 | 55 | 80 | 100 | 115 | 75 |
| Motor composite score | - | 82 | 89 | 85 | 116 | 79 | 73 | 92 | Not valid | 109 | 61 |
| Fine motor standard score | - | 11 | 10 | 8 | 13 | 7 | 8 | 9 | 10 | 12 | 4 |
| Gross motor standard score | - | 4 | 7 | 8 | 12 | 7 | 4 | 9 | Not valid | 11 | 4 |

Notes. IVH = intraventricular hemorrhage; CBH =cerebellar hemorrhage; WMI = grade of white matter injury; VD = ventricular dilatation; CP = cerebral palsy, GMFCS = Gross motor function classification system; ISCED = international standard classification of education with low (1 and 2), middle (3 and 4), and high (> 4) level.
