# Supplementary material for: Predictive utility of a simple cranial magnetic resonance imaging score at term‐equivalent age for cerebral palsy
Source: Dev Med Child Neurol. 2026 Mar 16;68(12):1729–39. doi: 10.1111/dmcn.70219 (PMC13743671; doi:10.1111/dmcn.70219)
Supplement: Supplementary file 4 [file DMCN-68-1729-s005.docx]

**Table S2.** Clinical characteristics of included (*n* = 137) and excluded (*n* = 122) very preterm infants due to missing cMRI and follow-up ≥ 18 months (corrected age).

|  | **Included infants**  **(*n* = 137)** | **Excluded infants**  **(*n* = 122)** | ***p*-Value** |
| --- | --- | --- | --- |
| Female, *n* (%) | 68 (50.4) | 56 (45.9) | 0.548 |
| Gestational age at birth in weeks, mean (SD) | 28.5 (2.5) | 29.2 (2.6) | 0.055 |
| Weight at birth in grams, mean (SD) | 1124 (0.4) | 1326 (0.4) | **< 0.001** |
| SGA, *n* (%) | 9 (6.6) | 5 (4.1) | 0.380 |
| Length at birth in centimeters, mean (SD) | 36.4 (4.6) | 38.5 (4.4) (*n*=119) | **< 0.001** |
| Head circumference at birth in centimeters, mean (SD) | 26.0 (3.0) | 27.4 (2.9) (*n*=119) | **< 0.001** |
| Antenatal betamethasone (2 applications at intervals of 24 hours), yes, *n* (%) | 122 (100) (*n*=122) | 94 (77.0) (*n*=106) | **< 0.001** |
| Inborn, yes, *n* (%) | 128 (93.4) | 108 (88.5) | 0.166 |
| Caesarean section, yes, *n* (%) | 121 (88.3) | 109 (89.3) (*n*=121) | 0.794 |
| 5-min APGAR score, mean (SD) | 7.9 (1.2) | 7.9 (1.5) (*n*=121) | 0.997 |
| 10-min APGAR score, mean (SD) | 8.6 (0.9) | 8.6 (1.0) (*n*=121) | 0.933 |
| Umbilical cord pH, mean (SD) | 7.30 (0.09) (*n*=132) | 7.30 (0.08) (*n*=115) | 0.911 |
| IVH, yes, *n* (%) | 33 (24.1) | 12 (9.9) (*n*=121) | 0.003 |
| IVH > grade II, yes, *n* (%) | 12 (8.8) | 7 (5.7) (*n*=121) | 0.361 |
| PVL, yes, *n* (%) | 13 (9.5) | 6 (4.4) (*n*=121) | 0.164 |
| BPD moderate/severe, yes, *n* (%) | 16 (11.7) | 7 (5.9) (*n*=119) | 0.106 |
| Treated BPD, yes, *n* (%) | 16 (11.7) | 11 (9.2) (*n*=120) | 0.512 |
| Hydrocortisone, yes, *n* (%) | 19 (13.9) | 12 (10.1) (*n*=119) | 0.119 |
| Pneumothorax, yes, *n* (%) | 8 (5.8) (*n*=136) | 4 (3.3) (*n*=121) | 0.328 |
| Amniotic fluid infection, yes, *n* (%) | 6 (4.4) | 7 (5.8) (*n*=120) | 0.595 |
| Postnatal sepsis, yes, *n* (%) | 55 (40.1) | 31 (25.4) (*n*=121) | **0.014** |
| Treated PDA, yes, *n* (%) | 52 (38.0) | 26 (21.3) (*n*=121) | **0.004** |
| Treated ROP, yes, *n* (%) | 9 (6.5) | 9 (7.4) | 0.799 |
| FIP, yes, *n* (%) | 2 (1.5) | 6 (4.9) (*n*=121) | 0.106 |
| NEC, yes, *n* (%) | 5 (3.6) | 7 (5.7) (*n*=121) | 0.416 |
| Neonatal seizures, yes, *n* (%) | 11 (8.0) | 6 (4.9) (*n*=120) | 0.330 |
| Surgeries in general, yes, *n* (%) | 28 (20.4) | 25 (20.5) (*n*=121) | 0.965 |
| Surfactant, yes, *n* (%) | 83 (60.6) (*n*=131) | 61 (50.0) (*n*=120) | **0.045** |
| Catecholamines, yes, *n* (%) | 16 (11.7) (*n*=136) | 13 (10.7) (*n*=120) | 0.814 |
| Parental ISCED, mean (SD) | 4 (2) (*n*=124) | 4 (2) (*n*=86) | 0.155 |

Notes: Data are presented as mean (standard deviation, SD) if not indicated otherwise. SGA = small for gestational age with weight of birth < third percentile; BPD = Bronchopulmonary dysplasia; treated BPD = hydrocortisone; PDA = patent ductus arteriosus; treated PDA = pharmacologic and/or surgical treatment; ROP = Retinopathy of Prematurity; treated ROP = pharmacologic treatment; FIP = focal intestinal perforation; NEC = necrotizing enterocolitis; ISCED = International Standard Classification of Education. Descriptive statistics are based on *n* = 137 for included and *n* = 122 for excluded infants, otherwise the number of cases is indicated in brackets.
